# Effect of COVID-19 on presentations of decompensated liver disease in Scotland

**DOI:** 10.1136/bmjgast-2021-000795

**Published:** 2022-01-06

**Authors:** Thomas Manship, Paul N Brennan, Iona Campbell, Stewart Campbell, Thomas Clouston, John F Dillon, Ewan Forrest, Andrew Fraser, Tee Lin Goh, Michael Johnston, Muhammad I Khan, Victoria Livie, Iain A Murray, Jayne Saunders, Debbie Troland, Ken J Simpson

**Affiliations:** 1 Centre for Liver and Digestive Disorders, Royal Infirmary of Edinburgh, Edinburgh, UK; 2 Centre for Regenerative Medicine, The University of Edinburgh Edinburgh Medical School, Edinburgh, UK; 3 Department of Gastroenterology, University Hospital Hairmyres, East Kilbride, UK; 4 The University of Edinburgh Edinburgh Medical School, Edinburgh, UK; 5 Gut Group, Ninewells Hospital and Medical School, Dundee, UK; 6 Division of Molecular and Cellular Medicine, University of Dundee School of Medicine, Dundee, UK; 7 Department of Gastroenterology, Glasgow Royal Infirmary, Glasgow, UK; 8 Department of Gastroenterology, NHSGGC, Glasgow, UK; 9 Dumfries and Galloway Acute Hospitals, Dumfries, Dumfries and Galloway, UK; 10 Department of Gastroenterology, Dumfries and Galloway Acute Hospitals, Dumfries, UK; 11 Gastroenterology, NHS Lanarkshire, Lanarkshire, Scotland; 12 Gastroenterology, NHS Greater Glasgow and Clyde, Glasgow, Scotland

**Keywords:** liver, COVID-19, alcoholic liver disease

## Abstract

**Background and aims:**

SARS-CoV-2 and consequent pandemic has presented unique challenges. Beyond the direct COVID-related mortality in those with liver disease, we sought to determine the effect of lockdown on people with liver disease in Scotland. The effect of lockdown on those with alcohol-related disease is of interest; and whether there were associated implications for a change in alcohol intake and consequent presentations with decompensated disease.

**Methods:**

We performed a retrospective analysis of patients admitted to seven Scottish hospitals with a history of liver disease between 1 April and 30 April 2020 and compared across the same time in 2017, 2018 and 2019. We also repeated an intermediate assessment based on a single centre to examine for delayed effects between 1 April and 31 July 2020.

**Results:**

We found that results and outcomes for patients admitted in 2020 were similar to those in previous years in terms of morbidity, mortality, and length of stay. In the Scotland-wide cohort: admission MELD (Model for End-stage Liver Disease) (16 (12–22) vs 15 (12–19); p=0.141), inpatient mortality ((10.9% vs 8.6%); p=0.499) and length of stay (8 days (4–15) vs 7 days (4–13); p=0.140). In the Edinburgh cohort: admission MELD (17 (12–23) vs 17 (13–21); p=0.805), inpatient mortality ((13.7% vs 10.1%; p=0.373) and length of stay (7 days (4–14) vs 7 days (3.5–14); p=0.525)).

**Conclusion:**

This assessment of immediate and medium-term lockdown impacts on those with chronic liver disease suggested a minimal effect on the presentation of decompensated liver disease to secondary care.

**Summary box**

**What is already known about this subject?**

- The COVID-19 pandemic has presented significant challenges to the provision of medical care for patients with liver disease in Scotland.

**What are the new findings?**

- Our data would suggest that in the short to medium term there did not appear to be any demonstrable adverse outcome in patients with chronic liver disease in Scotland.
- This finding included those with alcohol-related liver disease, which has been shown to have increased mortality in England and Wales over the course of the pandemic.

**How might it impact on clinical practice in the foreseeable future?**

- While our primary focus related to the initial lockdown period of 2020 was reassuring, it will be important to see the latter effect of further lockdown measures.
- Future work aimed at assessing the unknown longer term implications of lockdown and the changes in healthcare delivery approaches post-pandemic are paramount in determining future-proofing strategies.
- This is particularly pressing given the persistent threat posed by further waves of endemic SARS-CoV-2 variants.

## Introduction

SARS-CoV-2 and consequent COVID-19 pandemic has presented unique challenges across the globe. Most countries introduced severe restrictions on individual freedoms in an attempt to reduce COVID-19-associated mortality. Persons with liver disease represent a diverse spectrum of aetiologies; however, they share a relative immune dysfunctional state. Recently, a large cohort study has been published, which suggested that SARS-CoV2 mortality in all-cause liver disease was 32% (71% respiratory failure),^1^ which has enormous implications for such patients.

COVID-19 lockdown began in Scotland on 23 March 2020. This led to a closure of non-essential services including pubs, restaurants, and shops. All non-essential workers were encouraged to work from home.^2^ There was a shifting of the National Health Service (NHS) care with all elective work cancelled and the focus becoming inpatient, emergency care with increasing emphasis on COVID-19-infected individuals.

An Alcohol Change UK survey^3^ reported that during the UK lockdown (24 March 2020–4 July 2020) alcohol consumption was reduced in the majority but 20% of respondents increased their alcohol intake. In those patients with a history of alcohol misuse, 24% had increased alcohol consumption and 17% relapsed to drinking.^4^ Scottish Public Health Observatory^5^ data show that Scotland has one of the highest rates of chronic liver disease (CLD) deaths in Europe. The rate in Scotland in 2016 was 16.8 per 100 000. This is in comparison with the Netherlands where CLD mortality was 3.8 per 100 000. Alcohol-related liver disease (ArLD) is the most common cause of CLD in Scotland. Although difficult to estimate the number of patients in the community with ArLD, due to undiagnosed disease, in 2018/2019 there were 129.3 per 100 000 hospital stays with CLD due to alcohol. In 2018, Scottish patients with CLD were over four times as likely to die if they had ArLD when compared with all other causes of liver disease^5^ (13.1/100 000 vs 3.1/100 000).

Mahmud *et al*^6^ showed in a large study of veterans in the USA with a history of CLD that they were less likely to be admitted to hospital and were more unwell when admitted early in the COVID-19 pandemic when compared with previous years. Gaspar *et al*^7^ found contrasting data during the Portuguese state of emergency.

In view of Scotland’s high rate of CLD due to alcohol and the reported increase in alcohol consumption during lockdown, our aim was to describe first the effect of lockdown on all liver-related admissions in the short and medium terms and second the outcome of patients with ArLD in the medium term.

## Materials and methods

To determine the immediate effect of COVID-19 lockdown, data were collected from across Scotland on patients admitted with liver disease. Patients admitted to seven Scottish hospitals with a history of liver disease under the care of a gastroenterologist on dedicated gastroenterology or hepatology wards between 1 April and 30 April 2020 were included and compared with those admitted in the same period in 2017, 2018 and 2019. The Scottish sites were Royal Infirmary of Edinburgh (RIE) and Western General Hospital, Edinburgh; St John’s Hospital, Livingston; Glasgow Royal Infirmary and Queen Elizabeth University Hospital, Glasgow; Ninewells Hospital, Dundee; Hairmyres Hospital, Lanarkshire and Dumfries and Galloway Royal Infirmary.

To assess the medium-term effect of the healthcare reorganisation during the pandemic, data were collected on patients admitted to the RIE with a history of liver disease under the care of a hepatologist. RIE is a large, tertiary hepatology centre. Patients admitted between 1 April 2020 and 31 July 2020 were compared with those admitted in the same period in 2017, 2018 and 2019.

We included any patient admitted under the care of a gastroenterologist or a hepatologist with a history of CLD, regardless of admission diagnosis. We excluded those patients under the age of 18, patients admitted electively and those with a history of liver transplantation. No patients were admitted with COVID-19 infection at the time of writing this manuscript.

Variables collected were patient demographics, reason for admission, disease characteristics (history of hepatocellular carcinoma (HCC), history of ascites, presence of oesophageal varices and history of hepatic encephalopathy (HE)). Information on aetiology of liver disease was also collected and was split into ArLD and non-ArLD. Diagnosis was as determined by the patient’s usual clinician. Where the patient had more than one diagnosis, the primary diagnosis as per their usual clinician was used. Severity of liver disease on admission was defined by United Kingdom End-stage Liver Disease (UKELD)^8^ and Model for End-stage Liver Disease (MELD^9^ scores. Data were collected on socioeconomic deprivation using the Scottish Index of Multiple Deprivation (SIMD) (2020 update). This is a composite score of multiple factors (including income, employment, health, education, access to services, crime and housing) to determine deprivation in different parts of Scotland.^10^ The reason for admission was condensed into ‘decompensation’ (jaundice, ascites, gastrointestinal bleeding, HE and spontaneous bacterial peritonitis) and ‘non-decompensation’ (all other diagnoses) for analysis. Outcome measures were inpatient mortality and length of stay.

Demographics and baseline characteristics of the cohort were compared using the Pearson χ^2^ test for categorical variables. Continuous variables were compared using the Student’s t-test (parametric) or the Mann-Whitney U test (non-parametric). Categorical variables were recorded as numbers (percentages) and continuous variables as median (IQR). Data were analysed using SPSS V.24. Significant values were determined by a p value of equal to or less than 0.05.

## Results

### Immediate effect of lockdown: April 2020 (seven Scottish sites)

Three hundred forty-eight patients were admitted to seven hospitals in April in the years 2017 (n=123), 2018 (n=116) and 2019 (n=109) in comparison with 111 in the same period in 2020 (table 1). Patient demographics and levels of socioeconomic deprivation were similar in the two time cohorts. Most admission blood variables were also similar between the two groups; however, serum sodium was significantly lower in the pre-COVID (2017–2019) cohort compared with the 2020 cohort (135 (130–138) vs 137 (132–140); p=0.005). Consequently, UKELD score in the pre-COVID cohort was significantly higher (56 (52–60) vs 54 (50–60); p=0.005). No difference in the pre-2016 MELD (without sodium) was observed. Characteristics of cirrhosis were also similar apart from the presence of HCC which was more prevalent in the pre-COVID cohort (7.2% vs 1.8%; p=0.036). Aetiology of liver disease was also similar between the two groups (when comparing ArLD with non-ArLD diagnoses).

**Table 1:** A comparison of patients admitted between 1 April and 30 April 2020 to seven sites across Scotland with a history of chronic liver disease

		Reference range	2017–2019 (n=348) Pre-COVID	2020 (n=111) Lockdown	P value
Patient demographics	Age (years)		57 (49–65)	59 (47–68)	0.879
	Gender (male) (%)		208 (59.8%)	59 (53.2%)	0.218
SIMD	1 (most deprived)		161 (46.3%)	46 (41.4%)	0.422
	2		56 (16.1%)	21 (18.9%)	
	3		45 (13.0%)	15 (13.5%)	
	4		54 (15.6%)	13 (11.7%)	
	5 (least deprived)		32 (9.2%)	16 (14.5%)	
Admission characteristics	Hb (g/L)	115–165	105 (82–123)	104.5 (86.5–127)	0.553
	WCC (×10^9^)*	4–11	7.4 (4.9–10.7)	7.6 (5.2–11.5)	0.591
	Platelets (×10^9^)	150–400	114 (75–174)	121 (88–185)	0.495
	PT (s)	10.5–13.4	17 (14–21)	17 (14–20)	0.378
	Urea (mmol/L)	2.5–6.6	5.0 (3.1–8.6)	4.9 (3.3–8.6)	0.542
	Sodium (mmol/L)	135–145	135 (130–138)	137 (132–140)	0.005
	Creatinine (mmol/L)	50–98	69 (56–97)	72 (51–103)	0.894
	Potassium (mmol/L)	3.6–5	4.1 (3.6–4.5)	4.1 (3.5–4.5)	0.711
	Bilirubin (mmol/L)	3–21	50 (24–113)	42 (18–101)	0.097
	ALT (U/L)	10–50	29 (20–47)	32 (23–45)	0.568
	ALP (U/L)	40–125	157 (111–230)	141 (104–202)	0.071
	Albumin (g/L)	36–47	27 (22–31)	28 (23–33)	0.082
	MELD		16 (12–22)	15 (12–19)	0.224
	UKELD		56 (52–60)	54 (50–60)	0.005
	Admission hospital	GRI	83 (23.9%)	23 (20.7%)	
		QEUH	77 (22.1%)	34 (30.6%)	
		RIE	76 (21.8%)	22 (19.8%)	
		Lanarkshire	43 (12.4%)	10 (9.0%)	
		Ninewells	41 (11.8%)	11 (9.9%)	
		DGRI	22 (6.3%)	7 (6.3%)	
		WGH	6 (1.7%)	4 (3.6%)	
Inpatient outcomes	Length of stay (days)		8 (4–15)	7 (4–13)	0.131
	Inpatient mortality (%)		36 (10.3%)	9 (8.1%)	0.490
Disease characteristics	HCC (%)		25 (7.2%)	2 (1.8%)	0.036
	Ascites (%)		187 (53.7%)	51 (45.9%)	0.953
	Varices (%)†		206 (67.5%)	57.3 (60%)	0.074
	HE (%)		110 (31.6%)	42 (37.8%)	0.225
Aetiology of liver disease	ArLD (%)		263 (75.6%)	82 (73.9%)	0.718
	Non-ArLD		85 (24.4%)	29 (26.1%)	
Reason for admission	Decompensation (%)		267 (76.7%)	80 (72.1%)	0.320
	Non-decompensation (%)		81 (23.3%)	31 (27.9%)	

All values are median/IQR unless otherwise stated.

*Patients admitted to one trust did not have white cell breakdown results recorded; therefore, this information was excluded from this part of the analysis.

†Those with no previous endoscopies were excluded (n=65).

ALP, alkaline phosphatase; ALT, alanine transaminase; ArLD, alcohol-related liver disease; DGRI, Dumfries and Galloway Royal Infirmary; GRI, Glasgow Royal Infirmary; Hb, haemoglobin; HCC, hepatocellular carcinoma; MELD, Modified score for End-stage Liver Disease; PT, prothrombin time; QEUH, Queen Elizabeth University Hospital; RIE, Royal Infirmary of Edinburgh; SIMD, Scottish Index of Multiple Deprivation; UKELD, United Kingdom End-stage Liver Disease score; WCC, white cell count; WGH, Western General Hospital.

Outcome data were also similar between the two groups. Median length of stay was 8 days (4–15) in the pre-COVID cohort compared with 7 days (4–13) in the COVID-19 cohort (p=0.131). Inpatient mortality was also not statistically different between the two groups (10.3% (n=36) vs 8.1% (n=9); p=0.490).

### Medium-term effect of lockdown: April–July 2020 (RIE)

To determine if there was a medium-term effect on COVID-19 healthcare reorganisation, 284 patients admitted with CLD in the 4-month period in 2017 (n=82), 2018 (n=86) and 2019 (n=116) were compared with 89 in 2020 to the RIE (table 2). Patient demographics and social deprivation scoring were similar. Blood results on admission were also similar apart from serum sodium which was significantly lower in the pre-COVID cohort (134 (130–138) vs 136 (131–139); p=0.042). Severity of liver disease scores (MELD and UKELD) were similar between the two groups in contrast with previous. Disease characteristics were also similar between the two groups apart from the history of HE which was more prevalent in patients admitted in the pre-COVID cohort (35.9% (n=102) vs 21.3% (n=19); p=0.01). Aetiology of liver disease between the two groups was similar. Patients in the pre-COVID cohort were more likely to have been admitted with decompensated liver disease than in the COVID-19 cohort (82.7% (n=235) vs 70.8% (n=63); p=0.014).

**Table 2:** A comparison of patients admitted between April and July 2017–2019 to those in 2020 to the Royal Infirmary, Edinburgh with a history of chronic liver disease

		Reference range	2017–2019 (n=284) Pre-COVID	2020 (n=89) Lockdown	P value
Patient demographics	Age (years)		57.1 (49–67)	58.0 (48–68)	0.912
	Gender (male) (%)		46 (55.4%)	175 (61.6%)	0.096
SIMD	1 (most deprived)		70 (24.7%)	22 (24.7%)	0.791
	2		65 (23.0%)	19 (21.3%)	
	3		51 (18.0%)	17 (19.1%)	
	4		43 (15.2%)	12 (13.5%)	
	5 (least deprived)		54 (19.1%)	19 (21.4%)	
Admission characteristics	Haemoglobin (g/L)	115–165	108 (90–122)	106 (83.5–121.5)	0.494
	WCC (×10^9^)	4–11	8.0 (5.9–11.5)	7.6 (5.3–11.8)	0.401
	Neutrophils (×10^9^)	2–7.5	5.54 (3.90–8.73)	4.75 (3.25–8.15)	0.241
	Lymphocytes (×10^9^)	1.5–4.5	1.11 (0.73–1.65)	1.16 (0.78–1.78)	0.388
	Platelets (×10^9^)	150–400	114 (75–174)	124 (80–175)	0.442
	PT (s)	10.5–13.5	17 (15–22)	17 (15–20)	0.469
	Urea (mmol/L)	2.5–6.6	5.3 (3.5–10.2)	5.5 (3.1–10.2)	0.896
	Sodium (mmol/L)	135–145	134 (130–138)	136 (131–139)	0.042
	Creatinine (mmol/L)	50–98	73 (61–109)	76 (59–112)	0.988
	Potassium (mmol/L)	3.6–5	4.0 (3.6–4.4)	4.0 (3.5–4.5)	0.980
	Bilirubin (mmol/L)	3–21	53 (29–149)	60 (31–109)	0.646
	ALT (U/L)	10–50	32 (20–54)	34 (22–54)	0.533
	ALP (U/L)	40–125	178 (116–256)	164 (124–210)	0.179
	MELD		17 (12–23)	17 (13–21)	0.805
	UKELD		57 (53–62)	57 (53–60)	0.246
Inpatient outcomes	Length of stay (days)		7 (4–14)	7 (3.5–14)	0.525
	Inpatient mortality (%)		39 (13.7%)	9 (10.1%)	0.373
Disease characteristics	HCC (%)		26 (9.2%)	4 (4.5%)	0.158
	Ascites (%)		177 (62.3%)	54 (60.7%)	0.780
	Varices* (%)		173 (62.0%)	50 (61.0%)	0.866
	HE (%)		102 (35.9%)	19 (21.3%)	0.010
Aetiology of liver disease	ARLD (%)		237 (83.5%)	76 (85.4%)	0.663
	Non-ARLD		47 (16.5%)	13 (14.6%)	
Reason for admission	Decompensation (%)		235 (82.7%)	63 (70.8%)	0.014
	Non-decompensation (%)		49 (17.3%)	26 (29.2%)	

All values are median/IQR unless otherwise stated.

*Those with no previous endoscopies were excluded (n=12).

ALP, alkaline phosphatase; ALT, alanine transaminase; ArLD, alcohol-related liver disease; HCC, hepatocellular carcinoma; MELD, Modified score for End-stage Liver Disease; PT, prothrombin time; UKELD, United Kingdom End-stage Liver Disease score; WCC, white cell count.

Outcome data were similar between the two groups. Median length of stay in both cohorts was 7 days (p=0.525) (7 days (4–14) vs 7 days (3.5–14)). Inpatient mortality was not significantly different (13.7% (n=39) vs 10.1% (n=9); p=0.373).

Although RIE is a large, tertiary referral centre for liver disease, with a transplant unit the vast major of these patients were using the centre as their local hospital (93%) with very few coming from out of area (7%).

**Figure 1:**
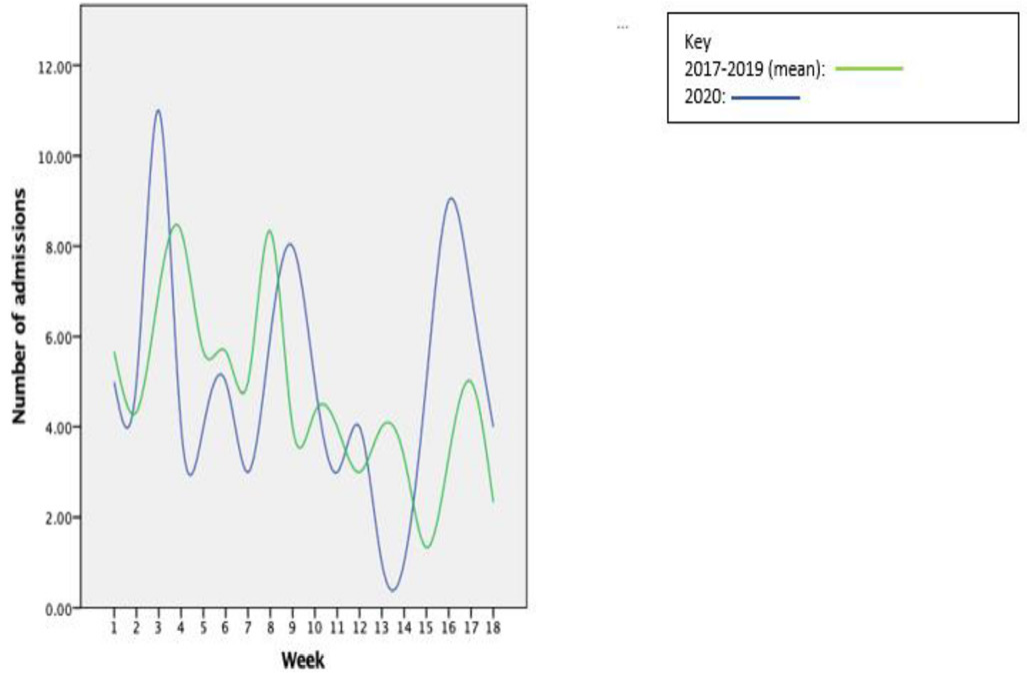
Weekly number of admissions in 2020 compared with the mean number of admissions in the previous 3 years.

We plotted the number of weekly admissions in the 18 weeks included in 2020 on the same graph as the mean number of admissions each week in 2017–2019 (figure 1). As can be seen, there are multiple points of crossover and therefore the weekly number of admissions are not significantly different.

As previously discussed, Scotland has a high rate of ArLD cirrhosis. Therefore, we performed a subgroup analysis as part of our medium-term cohort of patients admitted to RIE. We compared those with ArLD admitted in 2020 (n=76; 85.4% of all patients admitted) with those in the previous 3 years (n=237; 83.4% of all patients admitted). The two groups were again similar, and no differences were found in patient demographics, severity scoring, length of stay or inpatient mortality (see online supplemental information). Patients admitted in the pre-COVID cohort were, once again, found to have a significantly lower serum sodium on admission compared with those in the previous 3 years (133 (129–137) vs 136 (131–139); p=0.010). This then led to a higher UKELD in patients admitted in 2017–2019 in comparison with the COVID-19 cohort (58 (54–63) versus 57 (53–60); p=0.040) that was not replicated in MELD scores. Patients with a history of HE were more likely to be admitted in the pre-COVID cohort than those in the COVID-19 cohort (36.3% vs 21.1%; p=0.014). No differences were found in the number of patients with other disease characteristics. No differences were seen in the reasons for admissions between the two groups.

10.1136/bmjgast-2021-000795.supp1 Supplementary data

Outcomes, once again, were found to be similar between the two groups. Median length of stay was 7 days in both groups (7 days (4–15) versus 7 days (4–14); p=0.821). Inpatient mortality was 13.5% in the pre-COVID cohort and 6.6% in the COVID-19 cohort (p=0.104).

## Conclusion

Our data demonstrate no immediate or medium-term effect of NHS reconfiguration and lockdown in Scotland on numbers of admissions of patients with liver disease or adverse outcomes, including length of stay and inpatient mortality. There was no suggestion that patients were admitted with more severe or advanced liver disease or complications of their disease. Patient demographics and socioeconomic deprivation were no different between the two groups in the immediate lockdown period, in the medium term and in the ArLD subgroup. Overall, all blood parameters on admission were the same apart from serum sodium which reached a statistically significant difference but is of doubtful clinical relevance. Given the heavy weighting of serum sodium in the UKELD score, this is the likely reason for the higher scores in the immediate post-lockdown group and in the ArLD subgroup. No other components of the UKELD score were significantly different, and the original MELD score, which does not include serum sodium, were also no different.

On the whole, disease characteristics were the same between the two groups, although we did note higher rates of HCC in the pre-COVID April group and HE in the pre-COVID April–July and ARLD subgroup. These differences are difficult to explain but it could be related to restricted healthcare provision during COVID lockdown, that is, less surveillance for diagnosis of HCC and less support for people with known HE. Another possibility is the effect of social isolation from family and carers during the pandemic. Patients are commonly admitted with HE due to concerns from family and therefore reduced interactions could lead to a reduced number of admissions. In a study examining the impact of COVID-19 on the management of HCC, Amaddeo *et al*^11^ found that fewer patients were referred with index cases of HCC, and fewer had a first diagnosis of HCC during a 6-week period of the pandemic compared with 2019. This was likely related to the reduction in patient contact with health practitioners. Gandhi Met *et al*^12^ also demonstrated a reduction in the rates of HCC diagnosis (26.7%) in an Asia-Pacific population between February and May 2020 compared with the previous year. Both these groups were focused on the diagnosis of HCC, but both demonstrated the reduced number of presentations to health services with HCC, similar to the data presented here.

In the short-term and medium-term cohorts, we found no difference in aetiology of liver disease when comparing patients with ArLD to all other patients. We had believed that patients with ArLD would be more prominent in the COVID-19 era cohorts due to increased alcohol intake during lockdown; however, as previously discussed, this seems to not be the case. Patients could be avoiding admissions due to concerns regarding COVID-19 and in turn there could be a delayed effect with no differences seen within the timeframe of this study.

Reasons for admission (decompensated disease vs non-decompensated disease) were also generally similar. In the medium-term cohort, there were more people admitted with decompensated disease in 2017–2019.

Patient outcomes, in this study both length of stay and inpatient mortality, are clearly the factors that impact patients most. Our study demonstrated no difference in length of stay or inpatient mortality in any of the three groups that we investigated. This is reassuring and demonstrates that, in both the short and medium terms, inpatient medical care has not been affected by the pandemic.

The decreasing number of CLD admissions in Scotland over the last few years should be considered. According to Scottish Public Health Observatory data,^13^ there were 11 002 admissions (208.01 per 100 000 population) to Scottish hospitals in the financial year 2016/2017 compared with 10 917 (204.4 per 100 000) in 2017/2018 and 10 378 (193.18 per 100 000) in 2018/2019. This demonstrates around a 5% reduction in hospital admissions. There has also been a reduction in the number of CLD-related deaths at the same time: from 890 in 2016 to 873 in 2018. It is possible that given the number of admissions had been decreasing pre-COVID that the pandemic has had minimal effect when comparing admissions in 2020 compared with the previous 3 years. This is also possible with inpatient mortality, although CLD deaths have decreased less significantly.

In May 2018, minimum unit pricing (MUP) for alcohol came into force at 50 pence per unit in Scotland.^14^ An analysis in 2021 showed a 4%–5% decrease in ‘off-trade’ alcohol sales (purchased from a shop rather than a pub or restaurant) in the 12 months following the implementation of MUP^15^ and analysis into how this interacts with hospital-related admissions and outcomes is ongoing. It is possible that this decrease in sales and presumed consumption of alcohol has led to fewer complications of ArLD and therefore a reduced number of admissions than would normally be expected and therefore altering the impact of the pandemic. This is important given most of the patients in both our cohorts had ArLD. A Public Health Scotland report,^16^ however, found that there was no significant change in alcohol consumption levels during the first lockdown compared with the same period the previous year. Although, this was felt to be due to an increase in ‘off trade’ drinking offset by a decrease in ‘on-trade’ drinking. There was also no significant increase in the number of weekdays with early drinking (before 17:00) or solitary drinking occasions in a week.

These results differ from the largest study to date in this area (n=12 467). Mahmud *et al*^6^ demonstrated almost 160 fewer admissions than would be expected in veterans with decompensated cirrhosis during the pandemic by difference-in-difference analysis. They also found that those admitted later in the pandemic had a higher admission MELD and shorter length of stay which they conclude is likely due to a combination of patients being encouraged to stay at home and patients avoiding hospital and therefore only presenting when more unwell. A reduction in the number of cirrhosis admissions was also seen in a tertiary Chinese centre^17^ during the height of lockdown (21 January 2020 to 31 March 2020) but this was a much smaller study (n=414) than Mahmud. This pattern of reduced admissions but those admitted being more unwell has been shown in patients with heart failure^18 19^ and those with acute coronary syndromes.^20^

In contrast, Gaspar *et al*^7^ carried out a study in Portugal looking at trends in cirrhosis admissions in comparison to previous years (n=40 in 2020). They demonstrated a similar number of admissions, severity of liver disease parameters and length of stay. They also demonstrated no difference in in-hospital mortality but did find significantly reduced readmission rates in 2020. A German study by Zu Siederdissen *et al*^21^ found that although there was a reduction in the number of emergency admissions to the hospital, there was no difference in the number of patients with liver cirrhosis nor a difference in the severity of liver disease at presentation nor 30-day mortality.

Our study clearly has several limitations. We were unable to gather data from across the whole of Scotland when looking at the initial month following COVID-19 lockdown. However, we did gather data from health boards with most liver-related admissions in recent years^13^ (NHS Lothian, NHS Greater Glasgow and Clyde and NHS Lanarkshire). Our study also covers three of the four largest cities in Scotland (Glasgow, Edinburgh, and Dundee). Scotland had very low COVID-19 prevalence during the summer of 2020 (during June and July less than 30 cases/day^22^ with less than 1% of all tests positive) and normal services, such as outpatient clinics and elective admissions, started to resume. Therefore, given the snapshot interval (April 2020), it is possible that this was not long enough to effectively assess the impact.

Our analysis of the medium-term effects of lockdown covered a longer period (April–July 2020); however, it was only based at a single centre (RIE). This means that it does not have the breadth of looking at more Scottish sites. However, RIE is a large, tertiary referral centre for liver disease in Scotland, which is large enough to be representative of the wider population. As mirrored in the Scotland-wide data, NHS Lothian, where RIE is situated, had minimal cases in the summer months of 2020. Indeed, on only 1 day was there more than 10 recorded cases in a day in June (2 June 2020; 14 cases^22^).

It is, however, possible that the reduced admission rates due to decompensation in the Edinburgh cohort in 2020 could reflect delayed presentations. As outlined by Tapper *et al*,^23^ normal services resuming following the first wave could lead to significantly increased demand due to previously delayed care and fear of seeking medical attention. This is corroborated by Toyoda *et al*^24^ who showed that liver clinic visits, hepatoma surveillance and diagnostic imaging in patients with cirrhosis decreased significantly during COVID-19 lockdowns in three medical centres. This will inevitably lead to a delay in the recognition of decompensation and the diagnosis of HCC. Blach *et al*^25^ also demonstrated the potential effects of reduced hepatitis C elimination due to the pandemic on excess HCC cases and excess liver related deaths between 2020 and 2030. Clearly, such changes could alter medium-term inpatient length of stay and mortality, but have not been captured in our cohort given the relatively short follow-up period.

Our study only looked at patients admitted to gastroenterology or hepatology wards. It is possible that given the restructuring of hospital services during the pandemic, that patients were admitted to other clinical areas and managed by other medical specialties and therefore would not be included in this cohort. It is unlikely, however, that patients with decompensated disease, which comprises most patients, would be admitted under the care of another specialty without specialist gastroenterology support.

In conclusion, our data would suggest that the initial lockdown related to the COVID-19 pandemic had minimal effect on patients with CLD in Scotland. While the initial focus was on the first lockdown period, it will be important to determine the effect of further lockdowns as the pandemic continues, particularly as the country leaves a period of further restriction. Future work aimed at assessing the longer term implications of lockdown is paramount in future care provision in case of further pandemic threats.

## Data Availability

All data relevant to the study are included in the article or uploaded as supplementary information.
