# Supplementary material for: Effect of COVID-19 on presentations of decompensated liver disease in Scotland
Source: BMJ Open Gastroenterol. 2022 Jan 6;9(1):e000795. doi: 10.1136/bmjgast-2021-000795 (PMC8739538; doi:10.1136/bmjgast-2021-000795)
Supplement: Supplementary data [file bmjgast-2021-000795supp001.pdf]

		Reference range	2017-2019 (n=237) Pre-COVID	2020 (n=76) Lockdown	p-value
Patient demographics	Age (years)		57 (49-62)	58 (48-65)	0.690
	Gender (male) (%)		153 (64.6%)	40 (52.6%)	0.063
Admission characteristics	Haemoglobin (g/L)	115-165	107 (90-122)	104 (83-121)	0.388
	White cell count (x10^9)	4-11	8.2 (6.1-12.0)	7.6 (5.3-11.2)	0.106
	Neutrophils (x10^9)	2-7.5	5.61 (1.08-9.20)	4.85 (3.19-7.98)	0.086
	Lymphocytes (x10^9)	1.5-4.5	1.13 (0.75-1.72)	1.18 (0.79-1.95)	0.494
	Platelets (x10^9)	150-400	114 (74-170)	133 (84-187)	0.211
	Prothrombin time (seconds)	10.5-13.5	18 (15-23)	17 (15-21)	0.132
	Urea (mmol/L)	2.5-6.6	4.9 (3.1-9.3)	5.2 (2.9-9.4)	0.711
	Sodium (mmol/L)	135-145	133 (129-137)	136 (131-139)	0.010
	Creatinine (mmol/L)	50-98	70 (59-105)	75 (58-109)	0.806
	Potassium (mmol/L)	3.6-5	3.9 (3.5-4.4)	3.9 (3.3-4.4)	0.388
	Bilirubin (mmol/L)	3-21	68 (33-159)	61 (31-111)	0.270
	ALT (U/L)	10-50	34 (21-56)	33 (22-54)	0.998
	ALP (U/L)	40-125	180 (119-251)	164 (124-213)	0.170
	MELD		17 (13-23)	17 (13-21)	0.307
	UKELD		58 (54-63)	57 (53-60)	0.040
Inpatient outcomes	Length of stay (days)		7 (4-15)	7 (4-14)	0.821
	Inpatient mortality (%)		32 (13.5%)	5 (6.6%)	0.104
Disease characteristics	HCC (%)		18 (7.6%)	2 (2.6%)	0.124
	Ascites (%)		156 (65.8%)	47 (61.8%)	0.527
	Varices* (%)		147 (63.4%)	41 (57.7%)	0.394
	HE (%)		86 (36.3%)	16 (21.1%)	0.014
Reason for admission	Decompensation (%)		198 (83.5%)	56 (73.7%)	0.056
	Non-decompensation (%)		39 (16.5%)	20 (26.3%)	

Table 3: A table to compare admission characteristics 2020 in patients with ARLD cirrhosis and those in 2017-2019. All data presented as median and interquartile range unless otherwise stated. *Those who had not had a baseline endoscopy were excluded (n=10). All values are median/interquartile range unless otherwise stated. Abbreviations: WCC-white cell count; PT-prothrombin time; ALT- alanine transaminase; ALP-alkaline phosphatase; MELD-Modified score for End-stage Liver Disease; UKELD-United Kingdom End-stage Liver Disease score; HCC-hepatocellular carcinoma; ARLD-alcohol related liver disease.
